# ABO/Rh Blood Groups and the Temperament (Mizaj) Based on Traditional Persian Medicine Perspective: A Cross-sectional Study -

**DOI:** 10.31661/gmj.v12i.3062

**Published:** 2023-07-15

**Authors:** Mohammad Mahdi Parvizi, Mohadeseh Molayemat, Maryam Rezaee, Babak Shirazi Yeganeh, Thomas Rampp, Mohammad Hashem Hashempur

**Affiliations:** ^1^ Molecular Dermatology Research Center, Shiraz University of Medical Sciences, Shiraz, Iran; ^2^ Research Center for Traditional Medicine and History of Medicine, Department of Persian Medicine, School of Medicine, Shiraz; ^3^ Student Research Committee, Shiraz University of Medical Sciences, Shiraz, Iran; ^4^ Iranian Blood Transfusion Organization, Fars Provence Branch, Shiraz, Iran; ^5^ Department of Pathology, School of Medicine, Shiraz University of Medical Sciences, Shiraz, Iran; ^6^ Department of Internal and Integrative Medicine, Kliniken Essen-Mitte, Faculty of Medicine, University of Duisburg-Essen, Essen, Germany; ^7^ Research Center for Traditional Medicine and History of Medicine, Department of Persian Medicine, School of Medicine, Shiraz University of Medical Sciences, Shiraz, Iran

**Keywords:** ABO Blood-Group System, Persian Medicine, Temperament, Traditional Medicine

## Abstract

Background: Mizaj is a fundamental concept in traditional Persian medicine (TPM) that explains the physiological and psychological traits of human beings. This study aimed to investigate the association between the ABO/Rh blood groups and the Mizaj.Materials and Methods: This cross-sectional study was conducted on 18-60 years old individuals who visited the Iranian Blood Transfusion Organization, Shiraz Branch. We used Mojahedi’s Mizaj questionnaire to determine the Mizaj of the participants. The participants’ blood group and Rh were determined based on the antibodies against types A, B, and Rh antigens of their blood. Results: We assessed 308 individuals consisting of 174 (56.49%) male and 134 (43.51%) female. The results of our study showed a significant relationship between the AB blood group and the cold Mizaj in both simple and compound Mizaj categories (P=0.012, P=0.014, respectively). Logistic regression analysis revealed that people with the AB blood group were 2.88 times more likely to have cold Mizaj than people with the O blood group (OR=2.88, 95% CI: 1.16 to 7.15). In addition, those with the A blood group were found to be 54% less likely to have a dry Mizaj than those with the O blood group (OR=0.46, 95% CI: 0.23 to 0.92). There was no significant association between the participants’ Mizaj and their Rh blood group (P0.05).Conclusion: It seems that there was a significant relation between the AB blood group and cold Mizaj, as well as the A blood group and dry Mizaj. However, further studies are recommended with controlling the probable confounding factors.

## Introduction

Traditional Persian medicine (TPM) is one of the most ancient forms of traditional holistic medicine that is based on humoral medical theories [1][2][3]. Mizaj (temperament) is a fundamental TPM concept [4][5][6][7][8]. It is a phenotype-based categorization describing all physiological and psychological aspects of human beings and can also explain the reason for the difference in the diseases’s presentation and the response to treatment in each person [6]. Moreover, Mizaj derives from the interaction of contrary qualities of hotness, coldness, dryness, and wetness in elements [6][9]. Abnormal Mizaj or sue-Mizaj referred to the condition of imbalance between the Mizaj qualities and is classified into 9 general categories: hot, cold, dry, wet, cold-wet, hot-wet, hot-dry, cold-dry, and medium type [6][9][10]. All humans have sanguineous (Dam), phlegmatic (Balgham), bilious (Safra, yellow bile), and melancholic (Savda) humor depending on the dominant humor in the body [11][6]. 

The dominant humor determined which diseases the person is susceptible to, and which treatments are more effective. In other words, according to the concepts of TPM, Mizaj could be defined as the presence of the humor(s) quality accumulated in the body, according to the level of hotness and dryness. When there is an imbalance in the amount of humor in the body, people develop sue-Mizaj (dystemperament) [11][10][12]. Previous studies analyzed the association between the Mizaj and many physical and mental characteristics. For example, a recent study demonstrated a correlation between hot temperament and neuroticism [13]. 

It has also been shown that there is a significant correlation between hot and dry Mizaj and type 1 diabetes mellitus [14]. In addition, another study showed that non-melanoma skin cancer (NMSC) was associated with dry Mizaj [15]. Analysis of the association between body mass index and Mizaj showed that there is a significant relationship between the fat mass and cold-dry and hot-wet Mizaj [16]. Moreover, the results of a study showed a significant relationship between low body mass index (BMI<18) in teenage girls and dry Mizaj [17]. Determination of ABO antigen in humans blood is one of the most important, available, unchangeable, and valuable factors for determining the fundamental difference among humans genotypes which was discovered by Karl Landsteiner, a Viennese pathologist, in 1900 [18]. It was divided into four blood groups consisting of A, B, AB, and O according to the surface antigens of red blood cells [18][19]. Several studies revealed the association between the ABO blood group and some medical conditions including cancer [20][21] gastric ulcer[22], some infectious diseases including Plamodium falciparum malaria [23], helicobacter pylori gastritis [24], COVID-19 [25][26], as well as some psychological conditions including anxiety and stress [27][28]. There is also some significant evidence that adherence to diets specific to one’s blood type can reduce the risk of cardiovascular disease and improve overall health. The ‘Blood-Type’ diet advises individuals to eat according to their ABO blood type to improve their health and reduce the risk of chronic diseases [29]. Since the publication of the book "Eat Right for Your Type" by D’Adamo, there have been many controversial discussions about the relationship between ABO blood types, dietary habits, and human health [30]. 

However, there are few studies on the association between physical characteristics, blood type, and Mizaj [31][32]. The result of a study on 640 participants in 1930 showed a significant association between the blood group and Mizaj [31]. Another study in this regard investigated the association between 12 blood markers and Mizaj [32]. 

In contrast to previous studies, results showed no relation between the ABO and Rh alleles and the Mizaj [32]. There was only a significant association between the Lewis alleles and Mizaj [32].

To the best of our knowledge, there was little evidence of a correlation between the Mizaj and blood groups in humans. Therefore, this study aimed to analyze the prevalence of Mizajs among the Iranian population by considering the individual’s ABO/Rh blood groups and evaluating the relationship between blood group and Mizaj among the study participants.

## Materials and Methods

Study Design, Sample Size, and Participants

This cross-sectional study was conducted from the 1st of August to the 10th of October, 2020 at the Iranian Blood Transfusion Organization, Shiraz Branch (one of the main branches in Iran), Fars Province, Iran. The study population consisted of all voluntary blood donors, aged 18-60 years, who were referred to this organization. The convenience sampling method was used to enroll the participants in our study. Participants with contraindications of blood donation including taking anti-coagulants, having high-risk sexual behaviors or multiple sex partners, active seizure disease, significant pulmonary or cardiac diseases, hepatitis, syphilis, and addiction to intravenous drugs were excluded. Additionally, patients with kidney, hepatic, and thyroid disorders were excluded due to the possible effects of these diseases on the Mizaj. Before the enrollment, the study procedure was explained to all participants, and written informed consent was obtained from them.

According to the prevalence of dry Mizaj (25%) among Rh-positive participants in our initial pilot sampling, using the following formula, the sample size was estimated to be at least 288 with overall power of 95% at a significant level of 0.05. 

Ethics Statements 

The study protocol was approved by the Ethics Committee of Shiraz University of Medical Sciences (Ethics Code: IR.SUMS.MED.REC.1399.308). In addition, written informed consent was obtained from all participants before the enrollment. Moreover, participants were assured that their data would remain confidential to the researchers.

Data Collection 

The Mojahedi’s Mizaj Questionnaire was utilized to determine the Mizaj of participants [33]. This questionnaire contains ten questions (scored from 1 to 3, based on a three-point Likert scale). Eight of these questions are related to the hot/cold quality and two others are related to the wet/dry quality. Therefore, each participant had two scores, one for the hot/cold quality (ranges from 8 to 24) and the other for the wet/dry quality (ranges from 2 to 6). Participants with ≥19 scores in the hot/cold subscale were located in the category of hot Mizaj and those with ≤14 scores were categorized as cold Mizaj. In the dry/wet subscale, participants with a≥5 score had dry Mizaj and with ≤3 the score had wet Mizaj. The psychometric properties of this questionnaire were approved in the Iranian population. The reliability of this questionnaire was approved by Cronbach’s α coefficient of 0.71 in the Iranian population. Moreover, the sensitivity and specificity of this questionnaire for detecting the individual’s Mizaj were acceptable.

Also, the blood group information of the participants was determined from their blood samples by surveying the antibodies against type A and B, as well as Rh in the participants’ blood.

Statistical Data Analysis

Data analysis was performed using SPSS (IBM Corp. Released 2017. IBM SPSS Statistics for Windows, Version 25.0. Armonk, NY: IBM Corp.) and Stata software version 14.2 (StataCorp LLC, 4905 Lakeway Drive, College Station TX77845, USA). Qualitative and quantitative data were represented as frequency (percentage) and mean ± standard deviation, respectively. Kolmogorov-Smirnov test was used to determine the normality distribution of data. In addition, Chi-square and independent t-test were used to analyze the relationship between the variables. Furthermore, multiple logistic regression was performed to estimate the effect sizes with controlling the effect of participants’ gender on the results of the study. P<0.05 was considered statistically significant.

## Results

**Table:** Table Missing data for 3 cases; b. Missing data for 4 cases; c. Missing data for 6 cases

**Variables**		**ABO blood groups**	**P-value **	**Rh blood groups**	**P-value**
Total	A (N=84)	B (N=69)	AB (N=33)	O (N=122)		Rh positive (N=226)	Rh negative (N=82)	
**Age, mean±SD^ a^**	37.90± 10.06	38.42± 9.90	37.82± 9.64	37.25± 9.09	37.75± 10.73	0.944	38.17± 10.00	37.13± 10.25	0.427
**Gender, N (%)**									
** Male**	174 (56.49)	47 (55.95)	36 (52.17)	19 (57.58)	72 (59.02)	0.834	131 (57.95)	43 (52.44)	0.387
** Female**	134 (43.51)	37 (44.05)	33 (47.83)	14 (42.42)	50 (40.98)	95 (42.04)	39 (47.56)
**Marital status, N (%) ^b^**									
** Single **	98 (32.24)	20 (24.10)	28 (41.18)	9 (27.17)	41 (34.17)	0.134	67 (30.18)	31 (37.80)	0.207
** Married **	206 (67.76)	63 (75.90)	40 (58.82)	24 (72.73)	79 (65.83)	155 (69.82)	51 (62.20)
**Educational level, N (%)^ c^**									
**Under diploma**	22 (7.28)	4 (4.88)	2 (2.94)	3(9.68)	13 (10.74)	0.452	15 (6.76)	7 (8.75)	0.503
** Diploma**	96 (31.79)	31 (37.80)	21 (30.88)	11 (35.48)	33 (27.27)	72 (32.43)	24 (30.00)
**Bachelor degree**	145 (48.01)	35 (42.68)	38 (55.88)	14 (45.16)	58 (47.93)	103 (46.40)	42 (52.50)
**Master’s degree and higher**	39 (12.91)	12 (14.63)	7 (10.29)	3 (9.68)	17 (14.05)	32 (14.41)	7 (8.75)

**Table:** Table P-values were calculated using chi-square test by comparing each of temperament with other temperaments. P-value≤ 0.05 was considered significant.

** **		**ABO blood group**	**Rh blood group**
***Mizaj***	N (%)	A	B	AB	O	P value^*^	Rh positive	Rh negative	P value^*^
***Simple Mizaj types***
**Hotness quality **									
** Hot**	91 (29.55)	20 (23.81%)	22 (31.88%)	5 (15.15%)	44 (36.07%)	0.063	66 (29.20%)	25 (30.49%	0.888
** Moderate**	166 (53.90)	45 (53.57%)	41 (59.42%)	18 (54.55%)	62 (50.82%)	0.728	125 (55.31%)	41 (50.00%)	0.439
** Cold**	51 (16.56)	19 (22.62%)	6 (8.70%)	10 (30.30%)	16 (13.11%)	**0.012**	35 (15.49%)	16 (19.51%)	0.488
** Total**	308	84 (100%)	69 (100%)	33 (100%)	122 (100)		226 (100%)	82 (100%)	
**Wetness quality**									
** Wet **	107 (34.74)	27 (32.14%)	30 (43.48%)	10 (30.30%)	40 (32.79%)	0.387	73 (32.30%)	34 (41.46%)	0.139
** Moderate**	122 (39.61)	43 (51.19%)	22 (31.88%)	12 (36.36%)	45 (36.89%)	0.073	89 (39.38%)	33 (40.24%)	0.896
** Dry **	79 (25.65)	14 (16.67%)	17 (24.64%)	11 (33.33%)	37 (30.33%)	0.112	64 (28.32%)	15 (18.29%)	0.079
** Total**	308	84 (100%)	69 (100%)	33 (100%)	122 (100)		226 (100%)	82 (100%)	
***Compound Mizaj types***
** Cold and wet**	13 (4.22)	5 (5.95%)	3 (4.35%)	1 (3.03%)	4 (3.28%)	0.799	9 (3.98%)	4 (4.88%)	0.730
** Hot and wet**	31 (10.06)	7 (8.33%)	11 (15.94%)	1 (3.03%)	12 (9.84%)	0.193	22 (9.73%)	9 (10.98%)	0.743
** Hot and dry**	23 (7.47)	2 (2.38%)	6 (8.70%)	1 (3.03%)	14 (11.48%)	0.070	17 (7.52%)	6 (7.32%)	0.952
** Cold and dry **	11 (3.57)	3 (3.57%)	2 (2.90%)	3 (9.09%)	3 (2.46%)	0.328	9 (3.98%)	2 (2.44%)	0.519
** Hot **	37 (12.01)	11 (13.10%)	5 (7.25%)	3 (9.09%)	18 (14.75%)	0.439	27 (11.95%)	10 (12.20%)	0.953
** Cold **	27 (8.77)	11 (13.10%)	1 (1.45%)	6 (18.18%)	9 (7.38%)	**0.014**	17 (7.52%)	10 (12.20%)	0.200
** Wet **	63 (20..45)	15 (17.86%)	16 (23.19%)	8 (24.24%)	24 (19.67%)	0.801	42 (18.58%)	21 (25.61%)	0.177
** Dry **	45 (14.61)	9 (10.71%)	9 (13.04%)	7 (21.21%)	20 (16.39%)	0.454	38 (16.81%)	7 (8.54%)	0.069
** Moderate**	58 (18.83)	21 (25.00%)	16 (23.19%)	3 (9.09%)	18 (14.75%)	0.097	45(19.91%)	13 (15.85%)	0.421
**Total**	308	84 (100%)	69 (100%)	33 (100%)	122 (100%)		226 (100%)	82 (100%)	

**Table:** Table Odds ratio adjusted by gender of the participants; b. CI: confidence interval; *P-value≤0.05 was considered significant.

		**Hotness quality **
*Mizaj*	Blood group	Hot *Mizaj*	Moderate *Mizaj*	Cold *Mizaj*
ABO Blood Group	Odds ratio (95% CI^b^)	P-value	aOdds ratio^a^ (95% CI)	P-value	Odds ratio (95% CI)	p-value	aOdds ratio (95% CI)	P-value	Odds ratio (95% CI)	P-value	aOdds ratio (95% CI)	P-value
O	1		1		1		1		1		1	
A	0.55 (0.30 to 1.03)	0.063	0.56 (0.30 to 1.05)	0.070	1.12 (0.64 to 1.94)	0.698	1.11 (0.63 to 1.93)	0.725	1.94 (0.93 to 4.03)	0.077	1.92 (0.92 to 4.01)	0.082
B	0.83 (0.44 to 1.55)	0.560	0.87 (0.46 to 1.66)	0.659	1.42 (0.78 to 2.58)	0.253	1.39 (0.76 to 2.53)	0.285	0.63 (.23 to 1.70)	0.361	0.61 (0.23 to 1.65)	0.334
AB	0.32 (0.11 to 0.88)	0.027*	0.31 (.12 to 0.87)	0.027*	1.16 (0.54 to 2.51)	0.704	1.16 (0.53 to 2.51)	0.713	2.88 (1.16 to 7.15)	0.023*	2.88 (1.16 to 7.18)	0.023*
RH	RH positive	0.94 (0.54 to1.63)	0.827	0.90 (0.52 to 1.58)	0.722	1.24 (0.75 to 2.05)	0.409	1.26 (0.76 to 2.10)	0.364	0.76 (0.39 to 1.45)	0.402	0.77 (0.40 to 1.48)	0.435
		Wetness quality
*Mizaj*	Blood group	Wet *Mizaj*	Moderate *Mizaj*	Dry *Mizaj*
	Odds ratio (95% CI)	P-value	aOdds ratio (95% CI)	P-value	Odds ratio (95% CI)	P-value	aOdds ratio (95% CI)	P-value	Odds ratio (95% CI)	P-value	aOdds ratio (95% CI)	P-value
ABO Blood Group	O	1		1		1		1		1		1	
A	0.97 (0.54 to 1.76)	0.923	0.96 (0.53 to 1.75)	0.903	1.79 (1.02 to 3.15)	0.042*	1.79 (1.02 to 3.14)	0.044*	0.46 (0.23 to 0.92)	0.028*	0.46 (0.23 to 0.93)	0.030*
B	1.58 (0.86 to 2.90)	0.142	1.55 (0.84 to 2.85)	0.157	0.80 (0.43 to 1.50)	0.487	0.79 (0.42 to 1.48)	0.465	0.75 (0.38 to 1.47)	0.402	0.78 (0.39 to 1.52)	0.459
AB	0.89 (0.39 to 2.05)	0.787	0.89 (0.39 to 2.05)	0.780	0.98 (0.44 to 2.17)	0.956	0.98 (0.44 to 2.17)	0.951	1.15 (0.51 to 2.61)	0.741	1.16 (0.51 to 2.65)	0.726
RH	RH positive	0.67 (0.40 to 1.13)	0.137	0.68 (0.40 to 1.15)	0.150	0.96 (0.58 to 1.62)	0.891	0.97 (0.58 to 1.63)	0.918	1.77 (0.94 to 3.31)	0.077	1.73 (0.92 to 3.25)	0.091

## Discussion

The results of our study showed that there is a significant association between the AB blood group and the cold Mizaj. In addition, findings revealed that the most common Mizaj types in all ABO blood groups were moderate in both hotness and wetness qualities. This finding was in line with the study of Parvizi et al. which demonstrated that the prevalence of moderate Mizaj was higher than its other types in a normal population including university staff [13]. On the other hand, there was no significant association between the Rh blood group and the participants’ Mizaj.

It seems that the current study was the first study that analyzed the association between the Mizaj types and the blood groups in Iran. While, there were a few studies analyzing the association of the Mizaj types with the physical and mental characteristics of the individuals, as well as the association of ABO blood groups with the psychological characteristics of individuals. However, there were few studies assessing the relationship between the ABO/Rh blood groups and the Mizaj. A study conducted by Furukawa T. et al. in 1930 showed that there was a significant correlation between the ABO blood groups and the Mizaj [31]. The results of our study also showed a relation between the cold Mizaj and the ABO blood group [31]. 

According to the Seyed Mahtab Ali et al.’s study, conducted in Aligarh in New Delhi, the bilious Mizaj was the most common one among participants with B positive blood group [34]. Another study by Harburg et al. showed a significant association between the Lewis blood marker and the Mizaj. However, the association between the ABO/ Rh blood group and the different types of Mizaj was not significant. Furthermore, according to the results of this study, Males’ Anger and Impulsivity were strongly connected with the Lewis red blood cell phenotypes, while Females’ Sensation-seeking (a subscale of impulsivity) was significantly associated with the Lewis red blood cell morphologies [32]. Although the evidence showed that there was a significant relationship between Mizaj and personality type [13], analyzing this association was not considered in this study. It is necessary to consider that the various results among the Mizaj- related studies could be based on several factors including the ethnicity of the participants, the tools for determination of the Mizaj, and the different age ranges of the participants. 

In addition, one of the important factors in the determination of Mizaj from the TPM viewpoint is psychological and mental conditions [9][6]. A study by Yogeeshwaran et al. showed that there was no relationship between ABO blood groups and the level of stress among students [35]. While according to the study of Pisk et al. psychiatric disorders were more common in people with AB blood group compared to other blood groups [36]. Moreover, Kakathkar et al. found that children with AB blood group had a significantly higher level of dental anxiety compared to the children with other blood groups [27]. However, according to the TPM, Mizaj is an inherent feature in people, it should be considered that several factors including gender, age, place of residence, job and occupation, lifestyle, and food habits affect the Mizaj quality [37][38][39]. Therefore, it is probable that the individual’s Mizaj has been changed during their lifetime [9][10]. But, the blood group is one of the non-changeable characteristics of each person, so it seems that this could be considered as an initial factor with the potential capacity to affect the individual’s Mizaj.

Multiple logistic regression demonstrated a relationship between people’s Mizaj and their ABO blood group. In this regard, it seems that having both A and B antigens together in the blood makes that person up to 2.88 times more susceptible to cold Mizaj. On the other hand, it seems that having only the A antigen in the blood may be a protective factor against dry Mizaj. These results could be considered as a base for further studies to clarify more physiological aspects of this phenomenon. 

The distribution of the ABO (without considering RH) blood group in the Iranian population has been reported as % 38 for the O blood group, 33% for the A, % 22 for the B, and 7% for the AB in a previous study [40], which were similar to our study. Therefore, it seems that the participants of this study could be considered representative of the normal population of Iran. However, there were some limitations in our study. 

First, our study was a single-center study. So multi-center studies are recommended in this regard. Second, the ethnicity of the participants was not considered in this study. Ethnicity could probably be a confounding factor in our study. Therefore, considering the participants’ ethnicity is recommended for further studies. Next, the temperament of the individuals may be affected by several factors, especially nutrition, food habits, occupation, smoking, and psychological conditions which were not controlled in this study. Considering the probable effects of these lifestyle and environmental factors, it is recommended to repeat this study using related tools, questionnaires, and developed analytical models. Next, this study was conducted on healthy participants who were referred to the Iranian Blood Transfusion Organization, Shiraz Branch, so it could be possible that the results of this study may not apply to unhealthy individuals and patients with Sue-Mizaj. Finally, in this study, we only analyzed the relationship between the RBO blood groups and the Rh antigen. Future studies are proposed to consider other surface antigens of RBCs.

## Conclusion

This study showed that the cold Mizaj was significantly more common in participants with the AB blood group and it was less in participants with the B blood group compared to other blood groups. In contrast, there was no association between temperament and the presence of Rh antigen on the surface of red blood cells. Overall, this was a basic research to analyze the relationship between two individual phenotypes, one from modern medicine (ABO/Rh blood groups) and the other from the TPM concept (Mizaj). The practical key point of this study could be emerging a theory that manages the nutrition and food, occupation, the place for residency, or other factors. Depending on an individual’s blood type, Mizaj may help people improve their health, quality of life, and professional performance. More studies are recommended on this issue with controlling some probable confounding factors.

## Conflict of Interest

None.
